# Three-Dimensional Magnetoelectric Nanocomposite GelMA Hydrogels for Wireless Electrical Stimulation of Cardiac Cells

**DOI:** 10.1021/acsami.6c05467

**Published:** 2026-05-07

**Authors:** Angel Viteri, Carolina Vargas-Estevez, Samuele Colombi, Leonor Resina, Huan Tan, Jordi Sort, Maria-Pau Ginebra, Elisabeth Engel, Carlos Alemán, Jose García-Torres

**Affiliations:** † Biomaterials, Biomechanics and Tissue Engineering Group, Department of Materials Science and Engineering and Institute for Research and Innovation in Health (IRIS), Universitat Politècnica de Catalunya-BarcelonaTech (UPC), Av. Eduard Maristany 16, 08019 Barcelona, Spain; ‡ Barcelona Research Center in Multiscale Science and Engineering, Universitat Politècnica de Catalunya-BarcelonaTech (UPC), Av. Eduard Maristany 16, 08019 Barcelona, Spain; § Departament d’Enginyeria Química, EEBE, Universitat Politècnica de Catalunya, C/Eduard Maristany, 10-14, 08019 Barcelona, Spain; ∥ Departament de Física, Universitat Autònoma de Barcelona, Bellaterra 08193, Spain; ⊥ Catalan Institute of Nanoscience and Nanotechnology (ICN2), CSIC and BIST, Bellaterra 08193, Spain; # Institució Catalana de Recerca i Estudis Avançats (ICREA), Pg. Lluís Companys 23, Barcelona 08010, Spain; ∇ IMEM-BRT group, Department of Materials Science and Engineering, Universitat Politècnica de Catalunya-BarcelonaTech (UPC), 08019 Barcelona, Spain; ○ Institute for Bioengineering of Catalonia (IBEC), Barcelona Institute of Science and Technology (BIST), Baldiri Reixac 10-12, 08028 Barcelona, Spain; ◆ Centro de Investigación Biomédica en Red de Bioingeniería, Biomateriales y Nanomedicina (CIBER-BBN), Instituto de Salud Carlos III, 28029 Madrid, Spain

**Keywords:** core−shell nanoparticles, GelMA hydrogels, magnetoelectric nanocomposites, wireless electrostimulation, cardiac tissue engineering

## Abstract

Bioelectrical cues are essential for cardiac function and regeneration, yet current electrostimulation strategies rely on invasive electrodes that limit spatial control and clinical translation. Here, we report magnetoelectric nanocomposite hydrogels that combine core–shell CoFe_2_O_4_@BiFeO_3_ magnetoelectric nanoparticles (ME NPs) with a photo-cross-linked methacrylated gelatin (GelMA) network, enabling wireless electroactivity through externally applied magnetic fields within a soft, biomimetic three-dimensional scaffold. Structural and physicochemical analyses confirmed the successful synthesis of crystalline core–shell ME NPs with strong interfacial coupling, as demonstrated by transmission electron microscopy, X-ray diffraction, X-ray photoelectron spectroscopy, and magnetic hysteresis measurements showing exchange bias effects. Homogeneous incorporation of ME NPs within GelMA produced highly porous and interconnected hydrogels, as revealed by scanning electron microscopy and microcomputed tomography. The presence of nanoparticles reduced equilibrium swelling and refined pore architecture, suggesting increased effective cross-linking density and nanoparticle–polymer interactions. Mechanical testing showed soft elastomeric behavior with compressive moduli compatible with cardiac tissue. Under dynamic magnetic stimulation, magnetoelectric hydrogels significantly enhanced cardiac cell viability, proliferation, and morphological organization compared with pristine GelMA controls. After 10 days, the metabolic activity of cells cultured on GelMA-ME NP hydrogels under stimulation was approximately 3-fold higher than that of unstimulated GelMA. These results demonstrate that magnetoelectric hydrogels provide an effective platform for wireless electrostimulation, offering promising opportunities for cardiac tissue engineering and implantable bioelectronic therapies without wired electrodes.

## Graphical abstract

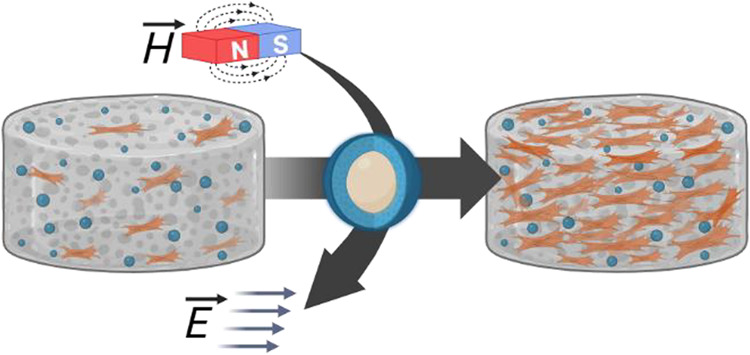

## Introduction

According to the World Health Organization, cardiovascular diseases are the primary causes of death worldwide with an estimated 19.8 million lives each year, representing approximately 32% of all the global deaths.^1^ This burden is expected to increase further over the following decades, as incidence and mortality rise globally in response to population growth and aging, posing a significant clinical challenge.^2^ Currently, cardiac transplantation represents the most effective treatment, but this medical procedure is limited by the heart availability, donor suitability and donor-receptor compatibility.^3^ The field of tissue engineering has emerged to respond to the global burden the lack of tissue and organ availability represents. Prove of its success is the fabrication and transplantation of some tissues -skin, bladder- ^4,5^ but little success has been achieved with more complex tissues with limited regenerative capacity like heart due to the challenge to replicate structural, vascular and functional properties.^3,6^

Recently, exogenous electric fields are being a promising strategy with high impact in tissue regeneration given the key role of electric fields in controlling myriad biological processes at tissue (e.g., neural communication, heart contraction, tissue repair after injury) and cell levels (proliferation, differentiation).^6,7^ Therefore, the development of smart material-based scaffolds capable of delivering wireless electrical stimulation to cells has recently garnered significant attention. A promising strategy involves exploiting piezoelectric materials, which generate surface polarization when subjected to mechanical strain, thereby serving as an effective means of electrostimulation. In this context, various piezoelectric materials, including collagen, zinc oxide, barium titanate, poly­(L-lactic acid) (PLLA), and poly­(vinylidene fluoride) (PVDF), have been explored for the fabrication of electroactive scaffolds. These mechanically responsive scaffolds have demonstrated great potential in enhancing cell adhesion, promoting proliferation, and facilitating differentiation.^8–10^

A second approach far less explored is the use of the magnetoelectric effect, or magnetoelectricity. Magnetoelectric effect refers to the phenomenon allowing for the conversion of an external magnetic field into a localized electric field (or *vice versa*) in a wireless and noninvasive way. This effect arises, for example, in magnetostrictive nanoparticles (e.g., Fe_3_O_4_, CoFe_2_O_4_) embedded into piezoelectric matrices like PVDF. In this line, different research groups have developed 2D piezoelectric polymeric films containing magnetostrictive nanoparticles for studying cell behavior under mechanical deformations.^11–14^ For example, Tang et al. have engineered a CoFe_2_O_4_/PVDF nanocomposite film exhibiting the ability to modify the surface potential in response to variable magnetic field strengths. Such surface potential variation in response to the external magnetic field modulated protein conformation and binding, enabling temporal dynamic responses of MC3T3-E1 preosteoblasts cells like adhesion, proliferation and osteogenic differentiation.^11^ Besides, Ribeiro et al. fabricated a Terfenol-D/PVDF composite film and seeded with MC3T3-E1 cells. The application of the magnetic field induced mechanical and electrical stimuli to the cells observing an increase in cell proliferation up to 25%, highlighting the effectiveness of ME stimulation in promoting bone tissue regeneration.^12^

Magnetoelectricity also arises in composite nanoparticles, typically composed of a combination of magnetostrictive and piezoelectric materials. During the application of a magnetic field, the magnetostrictive material deforms and the generated stress is transferred to the piezoelectric adjacent counterpart, which in turn becomes electrically polarized, thus, enabling the magnetoelectric nanoparticles (ME NPs) to generate electric fields when subjected to an external magnetic stimulus. ME NPs are very interesting as the strength of the magnetoelectric effect in nanomaterials is very high compared to bulk materials due to their larger surface to volume ratio.^15^ These ME NPs themselves are very promising in generating electric fields at the micro/nanoscale with high potential for electrically stimulating cells. Pané and coauthors developed two types of microrobotic platforms: one composed of gelatin-methacryloyl (GelMA) containing ME NPs (CoFe_2_O_4_@BiFeO_3_) and fabricated by two-photon polymerization and a second one consisting of SH-SY5Y neuroblastoma cell sphereoids loaded with ME NPs (CoFe_2_O_4_@BiFeO_3_). Both micromotors allowed to wirelessly and locally electrostimulate SH-SY5Y cells, favoring neuronal differentiation.^16,17^ These ME NPs have also been incorporated into 3D polymeric scaffolds (e.g., PLLA, PVDF) aiming to mimic the 3D and porous nature of extracellular matrix.^18,19^ Although those studies report promising results, these polymeric scaffolds have some drawbacks that limits their applicability in real tissue regeneration scenarios. First, the lack or limited biodegradability of the polymers used. PVDF is nonbiodegradable and it is therefore not so convenient for *in vivo* applications. Conversely, PLA is biodegradable, but its degradation rate is slow and inconsistent, potentially leading to accumulation of acidic degradation byproducts that may alter local pH and affect cell viability. Second, these polymers often exhibit limited cell adhesion and proliferation because they lack intrinsic bioactive sites and/or show hydrophobicity, which limits protein adsorption and reduces initial cell attachment. Third, they generally have high mechanical stiffness, which does not fully mimic the native extracellular matrix (ECM), potentially limiting their ability to support tissue integration. Fourth, while efforts have been made to improve porosity (e.g., inverse opal structures, nylon template-assisted fabrication), achieving the necessary pore size and interconnectivity for efficient nutrient and oxygen diffusion remains challenging. For example, pores formed through solvent casting in PVDF scaffolds can be nonuniform and small (5–20 μm), which may not fully support cell migration and vascularization.^19^ Finally, the synthesis procedures are neither biologically nor environmentally friendly due to the use of many organic solvents. Despite these advances, magnetoelectric polymeric scaffolds are primarily optimized for structural or actuation purposes rather than for biomimetic 3D cell culture.

In order to overcome those limitations, functional hydrogels represent a great alternative to develop biomimetic 3D scaffolds. Hydrogel-based systems offer a highly hydrated and bioactive microenvironment that more closely resembles native extracellular matrix. Moreover, conferring functional properties (e.g., electric, magnetic) to hydrogels have enabled great advances in a wide range of modern technologies, including bioelectronics, drug delivery, soft robotics, as well as tissue engineering and regenerative medicine.^20–23^ Thus, functional hydrogels offer a promising alternative to 3D polymeric scaffolds for tissue regeneration, owing to the inherent properties of hydrogels, such as high biocompatibility and bioactivity, hydrophilic nature – thereby enhancing protein adsorption leading to better cell adhesion, proliferation and differentiation–, tunable biodegradability, softness and flexibility – mimicking the mechanical properties of native extracellular matrix–, high porosity and permeability – facilitating oxygen and nutrient diffusion– as well as their ability to respond to external stimuli.^24,25^ Thus, in this work we report on the fabrication of 3D porous biocompatible and biodegradable hydrogels composed of methacrylated gelatin (GelMa) and core–shell ME NPs (CoFe_2_O_4_@BiFeO_3_) able to support cardiac cell electrostimulation through the application of external magnetic fields. GelMA hydrogels were selected as they provide intrinsic cell-adhesive motifs, tunable biodegradability, and soft mechanical properties compatible with cardiac tissue. On the other hand, and among the different magnetoelectric nanoparticle systems reported, CoFe_2_O_4_@BiFeO_3_ core–shell nanoparticles are particularly attractive due to the combination of strong magnetostrictive response from the CoFe_2_O_4_ core and piezoelectric behavior from the BiFeO_3_ shell, as well as the enhanced interfacial coupling enabled by the core–shell architecture, resulting in high ME coefficients, and their noncytotoxic behavior.^16,17,26,27^ Thus, these CoFe_2_O_4_@BiFeO_3_ containing GelMA hydrogels enable wireless, noninvasive and remotely controlled stimulation of electroactive cells without the need for rigid electrodes, making them an ideal component for next-generation cardiac regenerative therapies. Therefore, integrating magnetoelectric nanoparticles within hydrogel matrices represents a significant advancement toward biologically relevant, wireless electroactive scaffolds.

## Materials and Methods

### Materials

Iron­(III) chloride hexahydrate (FeCl_3_·6H_2_O), cobalt­(II) chloride hexahydrate (CoCl_2_·6H_2_O), bismuth­(III) nitrate pentahydrate (Bi­(NO_3_)_3_·5H_2_O), iron­(III) nitrate nonahydrate (Fe­(NO_3_)_3_·9H_2_O), cetrimonium bromide (CTAB), sodium hydroxide (NaOH), ethylene glycol, type B bovine skin gelatin, methacrylic anhydride, phosphate buffered saline (PBS), dialysis bags (12–14 kDa), and 2-hydroxy-4′-(2-hydroxyethoxy) −2-methylpropio phenone (Irgacure 2959), Dulbecco’s Modified Eagle Medium (DMEM) supplemented with 10% (v/v) fetal bovine serum and 1% (v/v) penicillin/streptomycin, 1% (v/v) l-glutamine were all acquired from ThermoFisher Scientific.

### Synthesis of Nanoparticles and Hydrogels

#### Core–Shell CoFe_2_O_4_@BiFeO_3_ Synthesis

ME NPs were synthesized with a core of a magnetostrictive cobalt­(II) ferrite (CoFe_2_O_4_) coated with a shell of piezoelectric bismuth­(III) ferrite (BiFeO_3_). Briefly, the core was fabricated following a hydrothermal process. First, an aqueous solution (deionized water) containing iron­(III) chloride hexahydrate (FeCl_3_·6H_2_O) (0.092 M), cobalt­(II) chloride hexahydrate (CoCl_2_·6H_2_O) (0.046 M) and cetrimonium bromide (CTAB) (0.07 M) was prepared to synthesize the core. Then, a 6 M sodium hydroxide (NaOH) solution was added to the previous solution under strong shaking to precipitate the CoFe_2_O_4_ NPs. This solution was sonicated for 2h, sealed in a Teflon-lined steel autoclave and heated at a temperature of 180 °C for 48 h. The core particles were collected by centrifugation, washed in ethanol and deionized water, and dried overnight at 80 °C. Later, the BiFeO_3_ shell was coated through a sol–gel process using as salt precursors bismuth­(III) nitrate pentahydrate (Bi (NO_3_)_3_·5H_2_O) (0.011 M) and iron­(III) nitrate nonahydrate (Fe (NO_3_)_3_·9H_2_O) (0.01 M) dissolved in ethylene glycol. Finally, 0.5 g of the CoFe_2_O_4_ core NPs were added to the above solution, mixed and left in a hot plate to dry overnight at 200 °C. The resulting CoFe_2_O_4_@BiFeO_3_ NPs were annealed at 600 °C, reached with a ramp of 10 °C/min, for 2 h.

#### GelMA Synthesis

Type B bovine skin gelatin in a concentration of 10% (w/v) was dissolved in distillated water at 60 °C under stirring (600 rpm). After that, the solution was cooled down to 50 °C and methacrylic anhydride was added dropwise until reaching a final concentration of 10% (w/v). The reaction continued for 4 h at 50 °C under stirring (300 rpm) to form the GelMA solution. The reaction was stopped by adding the equivalent volume of PBS. The solution was dialyzed at 40 °C for 7 days in a tank containing distilled water and using dialysis bags of 12–14 kDa cutoff (D9402, Sigma-Aldrich). The deionized water was kept in an agitation of 300 rpm and it was changed daily. The obtained GelMA solution was frozen at −80 °C overnight and lyophilized for 5 days. The final product was stored at −20 °C until further use.

#### Fabrication of the GelMA and GelMA-ME NPs Hydrogels

First, a 5% (w/v) GelMA aqueous solution was prepared followed by the addition of the photoinitiator 2-hydroxy-4′-(2-hydroxyethoxy)–2-methylpropio phenone (Irgacure 2959) until reaching a 0.5% (w/v) concentration. The GelMa solution was illuminated with a UV lamp of 365 nm (Analytic Jena UVP XX15 L) with a power intensity of 2.16 mW/cm^2^ for 180 s to chemically cross-link the methacryloyl groups and get the bare GelMA hydrogel. The hybrid GelMA-ME NPs hydrogel was prepared following the same procedure but adding the ME NPs in powder form to the initial GelMA-photoinitiator solution to have a final concentration of 10 wt % (regarding the hydrogel solid content). The resulting mixture was homogenize using a speed mixer (5 min, 3500 rpm) before UV irradiation. GelMA was dissolved in PBS to prepare hydrogels for the physicochemical characterization and in culture media for the biological studies.

### Material Characterization

The size, morphology and structure of the ME NPs were studied by transmission and high-resolution transmission electron microscopy (TEM, HRTEM) using a FEI Tecnai F20 microscope at a voltage of 200 kV. ME NPs were first ultrasonicated for 30 s in cycles of 10 s in ethanol. Then, a copper microgrid was dipped carefully in the above solution and left to dry at room temperature. Images were acquired and analyzed with Gatan microscopy suite software. Fast Fourier transform (FFT) analyses were also performed with the same software. Particle size distributions were obtained by analyzing the TEM micrographs using ImageJ software (version 1.54g), with a total of 225 ME NPs analyzed to ensure a representative data set.

The crystallographic structure of the core–shell ME NPs was analyzed by X-ray diffraction (XRD) on a Bruker AXS D8 Advance X-ray diffractometer in a standard Bragg–Brentano configuration. Cu Kα radiation (λ = 1.5406 Å) was employed with operating conditions set at 40 kV and 40 mA. Data acquisition was performed with a fixed step size of 0.02° and a measurement duration of 1 s per step, ensuring high-resolution analysis for phase identification and evaluation of the crystalline structures present in the ME NPs.

X-ray photoelectron spectroscopy (XPS) analyses were performed on a SPECS system. The spectrometer featured a high-intensity twin-anode X-ray source (XR50) with Mg/Al (1253/1487 eV), operating with the Al anode at 150 W. The X-ray source was positioned perpendicular to the analyzer axis, and a Phoibos 150 MCD-9 XP detector was used for detection. The stage position was digitally controlled to acquire data at three different spots of the samples. The spectra were recorded at 25 eV, and high-resolution spectra were recorded with an energy step of 0.1 eV. The analysis chamber pressure was kept below 10^–7^ Pa and binding energies (BE) of the XPS signals of all species were referenced to the C 1s signal at 284.6 eV.^28^ CasaXPS software (Casa Software Ltd., Devon, UK) was used for the peak’s deconvolution.

Proton nuclear magnetic resonance (^1^H NMR) was performed with a Bruker Ascend-400 spectrometer operating at 400.1 MHz and recording 128 scans. The chemical shift was calibrated using tetramethylsilane as internal standard. The spectra of gelatin and GelMA were obtained after dissolving them in deuterated water by applying sonication for 24 h and at 50 °C (Gelatin) and room temperature (GelMA). The degree of methacrylation (DoM) was calculated taking into account the decrease in the integrated signal of lysine methylene of gelatin after methacrylation.^29^

The surface morphology of the hydrogel was examined using a Zeiss Neon 40 analytical field emission scanning electron microscope (FESEM). Prior to imaging, the samples underwent lyophilization and were subsequently affixed to an aluminum stub and coated with gold via sputtering. Observations were conducted at an applied voltage of 15 kV. Additionally, energy-dispersive X-ray (EDX) analysis was carried out with the same microscope to detect the presence of the different materials.

The porosity of the lyophilized hydrogels was investigated by X-ray microtomography images (micro-CT) with a Skyscan 1272 Bruker microCT system (Kontich, Belgium). Scans were performed at a voltage of 50 kV and a current of 200 μA, without a filter. Projected images were captured every 0.2° over a full 360° rotation, with an exposure time of 931 ms, resulting in an isotropic voxel resolution of 3 μm. Sample reconstruction was conducted using nRecon software (Bruker), which involved alignment adjustments, beam hardening correction, and ring artifact removal. Porosity quantification was carried out with CTAn software (Bruker) by applying binary segmentation, eliminating speckles smaller than 15 voxels, and performing measurements. Additionally, CTvox software (Bruker) was utilized to generate 3D visualizations of the reconstructed structures.

Raman spectroscopy was performed using a Renishaw inVia Qontor confocal Raman microscope. The system included a 785 nm laser with a nominal output power of 500 mW, which was focused onto the sample through a Leica DM2700 M microscope. The scattered light was then collected and directed to a spectrometer equipped with a 1200 lines·mm^–1^ grating. Spectral acquisition was conducted with an exposure time of 10 s, using a laser power set to 1% of its nominal output, and each spectrum was obtained by averaging three accumulations to enhance signal quality.

The hydrogel equilibrium swelling ratio (SR) was measured gravimetrically in water at room temperature. Hydrogels were lyophilized overnight before recording their dry weight (*W*_d_). They were then submerged in deionized water for 24 h to reach equilibrium. After immersion, excess surface water was gently removed using filter paper, and the swollen hydrogels were weighed (*W*_w_). The SR was determined using the following eq (eq 1)­

1 $$\mathrm{SR}=\frac{W_{w}-W_{d}}{W_{d}}$$

The weight was determined with an analytical balance with 0.01 mg resolution. To ensure reproducibility, three independent measurements were conducted.

Magnetometry measurements were performed using a Microsense EZ vibrating sample magnetometer (VSM) at room temperature. A precise analytical balance was used to weight a small amount of dried powder, which was then compacted into a nonmagnetic capsule for hysteresis loop measurements. Hysteresis loops were recorded both prior to heating (as-grown state) and immediately after heating the samples to 650 K and cooling them under an in-plane magnetic field (*H* = 10 kOe) (field-cooled state). This temperature lies just above the Néel temperature of BiFeO_3_. The field-cooling procedure was employed to probe whether the interface between the CoFe_2_O_4_ cores and BiFeO_3_ shells is of sufficient quality to allow mutual exchange coupling between the cores and shells within the nanoparticles.^30^ Hysteresis loops were recorded with a maximum applied magnetic field of 20 kOe. An integration time of 1 s was used to have an adequate signal/noise ratio. High-field linear background signals, arising from the sample holder and substrate, were subtracted.

The mechanical properties of the hydrogels were evaluated using a hybrid rheometer (Discovery HR-2, TA Instruments) operating in compression mode. Cylindrical hydrogel samples (18 mm diameter) were subjected to uniaxial compression up to 75% strain at a constant displacement rate of 7.5 μm s^–1^ at room temperature. Stress–strain curves were recorded, and the compressive elastic modulus was calculated from the slope of the linear region of the curve corresponding to 10–20% strain.

All measurements were performed in triplicate.

### Magnetic Stimulation Set Up

The magnetic actuation system used for cell stimulation consisted of a rotating Teflon disk embedding 16 permanent cylindrical magnets arranged symmetrically along its circumference. Rotation of the disk at a controlled frequency generates a time-varying (alternating) magnetic field at the position of the cell culture plate, located over the magnet disk. The magnetic field amplitude was set with the magnet grade and the distance between the magnetics and the culture surface. In this setup, neodymium–iron-boron (NdFeB) permanent magnets (grade N52; diameter 10 mm; thickness 5 mm; Supermagnete) were used and positioned at a fixed distance of 2 mm from the culture plate, producing a magnetic field strength of 150 mT at the sample location. The magnetic field intensity was experimentally quantified using a teslameter (FM 302, Projekt Elektronik GmbH, Berlin, Germany). The rotation frequency of the magnet disk, and thus the magnetic stimulation frequency, was controlled using a digital tachometer (DT-2234C, Topway).

### Biological Characterization

Cell viability and proliferation assays were performed using H9c2 rat cardiac myoblasts. Cells were cultured in Dulbecco’s Modified Eagle Medium (DMEM) supplemented with 10% (v/v) fetal bovine serum,1% (v/v) penicillin/streptomycin, and 1% (v/v) l-glutamine and maintained at 37 °C in a humidified atmosphere with 5% CO_2_. Prior to cell seeding, hydrogel samples were sterilized under UV light for 30 min and preconditioned with DMEM for an additional 30 min. After removal of the DMEM, a 10 μL droplet containing 20.000 cells per sample was seeded onto each hydrogel. Samples were incubated for 30 min to allow initial cell settling and attachment, followed by the careful addition of fresh culture medium. Following cell seeding, samples were cultured either under static conditions or under dynamic magnetic stimulation. Magnetic stimulation was applied using the above-described setup, with a magnetic field strength of 150 mT and a frequency of 500 Hz for 1 h per day (30 min in the morning and 30 min in the afternoon).

Cell viability was assessed at days 1, 7, and 10 using a Live/Dead fluorescence staining kit (Invitrogen). At each time point, DMEM was removed and samples were incubated with 500 μL of Live/Dead staining solution for 30 min at room temperature. Subsequently, samples were rinsed with phosphate-buffered saline (PBS) to remove excess dye and imaged using a confocal microscope (Carl Zeiss LSM 800). Images were acquired using Zen software (version 2.6, Blue edition).

Cell proliferation was evaluated using the PrestoBlue assay (Invitrogen) at days 1, 3, 7, and 10. Prior to fluorescence measurements, samples were transferred to a new well plate to ensure that the recorded signal originated exclusively from cells. Hydrogels without cells were included as blank controls. Samples were then incubated with PrestoBlue reagent diluted in fresh DMEM for 1 h at 37 °C. After incubation, 100 μL of the supernatant was transferred to a black 96-well plate, and fluorescence was measured using a Tecan Infinite 200Pro microplate reader and Tecan i-control software (version 3.9.1.0), with excitation and emission wavelengths set to 560 and 590 nm, respectively. The number of cells was determined using the fluorescence calibration curve (Figure S1), which was obtained by plotting fluorescence against a known number of cells. Experiments were performed three times with three replicates each experiment.

Statistical analysis was performed using GraphPad Prism (version 9.3.1). Data normality was assessed using the Shapiro–Wilk test. Statistical comparisons were conducted using two-way analysis of variance (ANOVA), followed by Tukey’s multiple comparisons test. A significance level of α = 0.05 was applied. Data are presented as mean ± standard deviation.

## Results and Discussion

### Conceptualization, Design and Synthesis of the Magnetoelectric Hydrogel

Electric fields in the human body play a key role in a myriad of biological processes like the well-known action potentials of nerves, muscles or heart, but also in controlling cellular features and functions, such as morphology, proliferation, gene expression or differentiation.^31^ Bioelectrical circuits and their wiring act as long-range intercellular signaling and controlling mechanisms in the development, maintenance or regeneration of tissues.^32,33^ Regarding heart, precise electrical signals are needed to coordinate its rhythmic contractions, ensuring efficient blood circulation throughout the body. Any disruption in this electrical activity can lead to arrhythmias or cardiac dysfunction, which highlights the critical role of bioelectrical stimulation in maintaining heart function.

Based on the importance of electric fields, we have designed a new hydrogel integrating ME NPs as a noninvasive and electrically active scaffold for cardiac tissue engineering. The composite hydrogels combine the ECM-like attributes of hydrogels (e.g., softness, high water content, biocompatibility) to support the cardiac cells, and the localized electric field generated by the ME NPs in response to external magnetic stimuli. Thus, this hybrid hydrogel is expected to provide multiple advantages: (i) wireless bioelectric stimulation (i.e., by applying an external magnetic field, ME NPs generate local electric fields that can stimulate cardiomyocyte activity), (ii) enhanced cell proliferation behavior (i.e., the presence of ME NPs can promote cell adhesion and/or proliferation, critical for tissue regenerating), and (iii) tunable mechanical properties (by adjusting hydrogel’s composition, the stiffness can be tuned to match native myocardial tissue).

The hydrogel is formed by cross-linking GelMA polymer under UV irradiation. ME NPs previously synthesized are present during the cross-linking so they are physically entrapped and homogeneously embedded into the hydrogel. The overall procedure for the preparation of the hybrid hydrogels involved the following steps. First, the hydrogel precursors – ME NPs, GelMA– were obtained (see experimental section for details). Briefly, core–shell CoFe_2_O_4_@BiFeO_3_ NPs were synthesized by a three-step process: (i) synthesis of the CoFe_2_O_4_ core via hydrothermal synthesis, (ii) coating the core with the BiFeO_3_ shell following a sol–gel procedure, and (iii) annealing of the NPs to crystallize them. Simultaneously, GelMA was obtained from gelatin through a chemical modification process involving methacrylation, dyalisis and lyophilization After that, an aqueous solution of GelMA and the photoinitiator was prepared followed by the addition of the ME NPs (the final content of the ME NPs was 10 wt % regarding the hydrogel solid (GelMA) content). Finally, GelMA cross-linking took place under UV irradiation.

### Characterization of the ME NPs

The morphology of ME NPs in their powder form was studied using transmission electron microscopy (TEM). Figure 1A presents a TEM image of the synthesized core–shell CoFe_2_O_4_@BiFeO_3_ NPs revealing that they are relatively uniform in size. A statistical particle size distribution analysis obtained from TEM images is provided in Figure S2. The resulting distribution was fitted with a log-normal function, yielding a mean diameter of 18.7 nm and a particle size distribution ranging from 4 to 50 nm. Moreover, core–shell formation can be inferred from highly underfocused micrographs (Figure S3), where nanoparticle contrast is enhanced, as reported by Crisan et al.^34^ Under these conditions, the presence of a darker core surrounded by a brighter shell provides evidence of the core–shell architecture of the synthesized nanoparticles. The shell thickness is locally estimated to be on the order of ∼1 nm. This estimation is consistent with the detection of Co in the XPS analysis of the ME NPs (see below), since the XPS probing depth (∼2 nm) is greater than the thickness of the BiFeO_3_ shell, allowing signal contribution from the core. Higher magnification TEM image (see inset Figure 1A) indicates that the particles are not spheres but nanooctahedrons. These faceted morphologies are particularly relevant, as they may facilitate interactions between the GelMA polymer chains and the ME NPs through the edges of the different faces, as we have previously observed in nanocomposite hydrogels [20]. In order to go deeper in the structural ME NPs characterization, high-resolution TEM (HRTEM) analysis was performed to reveal the crystalline symmetry and phase distribution of CoFe_2_O_4_ and BiFeO_3_ materials (Figure 1B). Multiple lattice fringe orientations can be observed indicating the polycrystalline nature of the NPs. Fast Fourier Transform (FFT) analysis was performed on different regions of the HRTEM image to confirm the distribution of the phases (Figure S4). The FFT analysis performed in the NPs shell exhibited an interplanar *d*-spacing of 0.2791 nm, which corresponds to the (110) plane of the rhombohedral structure of the BiFeO_3_ shell (space group *R3c*). Meanwhile, FFT analysis conducted on the NPs center, including both core and shell regions, mainly identified *d*-spacings attributed to the spinel ferrite face-centered cubic lattice (FCC) of the CoFe_2_O_4_ (spatial group *Fd3̅*), as expected, due to the higher proportion compared to the BiFeO_3_. Thus, the following *d*-spacings were identified 0.4867, 0.2553, and 0.2118 nm attributed to the (111), (311) and (400) planes. A *d*-spacing of 0.3895 nm was also identified that could correspond to the (012) plane of the rhombohedral-BiFeO_3_. The *d*-spacings were assigned based on the lattice parameter data provided in the corresponding powder diffraction files for CoFe_2_O_4_ (ICSD 00–022–1086) and BiFeO_3_ (ICSD 01–086–1518). Based on these findings it can be concluded that CoFe_2_O_4_ is predominantly localized in the central region, while BiFeO_3_ is concentrated toward the edges of the nanoparticle, confirming the formation of the core–shell structure. Furthermore, the observed multiple lattice orientations and the crystallographic mismatch between CoFe_2_O_4_ and BiFeO_3_ indicate that the shell is not epitaxially grown but rather forms a polycrystalline coating with interfacial contact.

**Figure 1:**
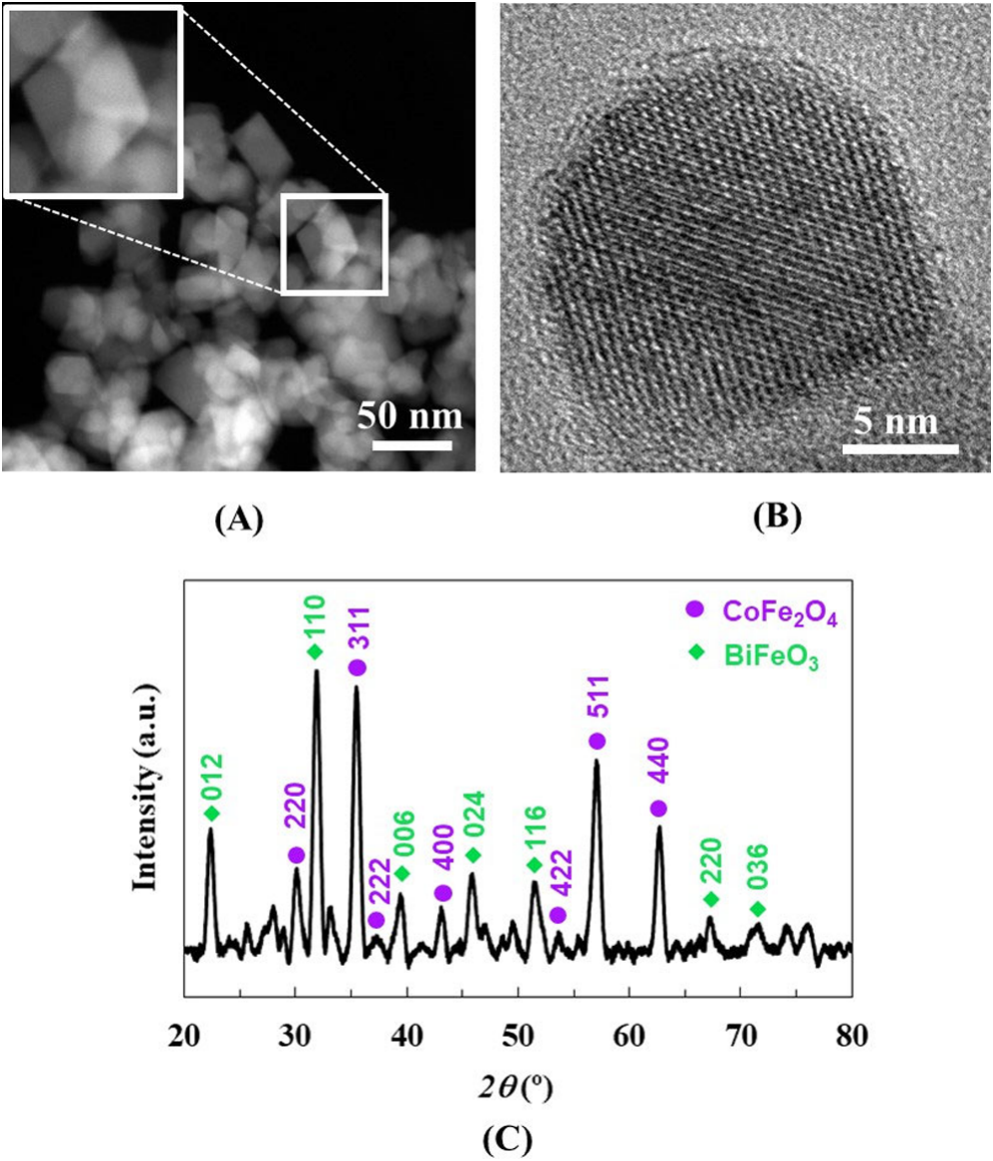
Characterization of CoFe_2_O_4_@BiFeO_3_ ME NPs: (A) TEM image and higher magnification TEM image (inset). (B) HRTEM image. (C) XRD pattern.

Figure 1C shows the X-ray diffraction (XRD) pattern of the core–shell NPs where well-defined peaks are observed, indicating once again their crystalline nature. The diffraction pattern exhibits characteristic peaks for both CoFe_2_O_4_ core and BiFeO_3_ shell phases. While peaks at 30.1, 35.5, 37.5, 43.1, 53.7, 57.1, and 62.7° correspond to the (220), (311), (222), (400), (422), (511), and (440) planes of the spinel ferrite FCC lattice of CoFe_2_O_4_, peaks at 22.4, 31.9, 39.4, 45.8, 51.5, 67.2, and 71.5° correspond to the (012), (110), (006), (024), (116), (220), and (036) planes of the rhombohedral perovskite lattice of BiFeO_3_. Additional reflections attributable to common secondary phases are not detected. These findings are consistent with those previously obtained through HRTEM analysis and with the literature.^18^

X-ray photoelectron spectroscopy (XPS) was performed to obtain a more precise understanding of the oxidation states of bismuth, iron and cobalt in the synthesized core–shell CoFe_2_O_4_@BiFeO_3_ NPs. The high-resolution narrow-scan XPS spectra are presented in Figure 2. The XPS survey spectrum reveals the presence of Fe, Co, Bi, and O, confirming the presence of both phases in the composite NPs. The presence of C 1s in the survey spectrum can be attributed to a combination of adventitious carbon contamination and residual carbon species originating from the synthesis route (e.g., ethylene glycol), which may not be completely removed during annealing. Since C 1s signal is very low compared to the others, it indicates that the resulting NPs are highly pure. The Bi 4f high-resolution spectrum shows two peaks at 160.5 and 165.8 eV, which are attributed to the Bi 4f_7/2_ and Bi 4f_5/2_ transitions, respectively, with a spin–orbit splitting of 5.3 eV. These results are consistent with the Bi­(III) oxidation state. On the other hand, the presence of Co from the CoFe_2_O_4_ core is attributed to the XPS beam’s penetration depth of approximately 2 nm, which exceeds the thickness of the BiFeO_3_ shell. The high-resolution Co 2p spectrum displays two main peaks at 782.1 eV (Co 2p_3/2_) and 797.5 eV (Co 2p_1/2_), accompanied by their characteristic satellite peaks at 787.6 and 805.3 eV, respectively. A binding energy separation between the main peaks of 15.4 eV is characteristic of Co­(II) oxidation state, which is typical for CoFe_2_O_4_ spinel structures. Moreover, the BE difference between the main peak and the satellite is also an indicator of the oxidation state of the cobalt ion. A narrow separation of about 4–6 eV is characteristic of Co­(II), while a larger difference of about 9–10 eV is often found with Co­(III).^35^ Since the separations between the main peaks and the corresponding satellites are 7.8 eV for Co 2p_3/2_ and 5.5 eV for Co 2p_1/2_, we can confirm that Co ions in cobalt ferrite are divalent. The slightly larger peak shift between the 2p_3/2_ peak and its satellite may be explained by the interaction with the BiFeO_3_ shell. Finally, it is also important to highlight that the intense satellite structure at the high energy binding side of the Co 2p_3/2_ and 2p_1/2_ transitions indicates that the majority Co^2+^ cations occupy octahedral sites in the CoFe_2_O_4_ spinel lattice.^36^ The Fe 2p core–electron spectrum exhibits two main peaks at 712.7 and 726.3 eV corresponding to Fe 2p_3/2_ and Fe 2p_1/2_, respectively, with an energy spin–orbit splitting of 13.6 eV. Two satellites are also observed at 720.1 and 734.2 eV attributed to Fe 2p_3/2_ and Fe 2p_1/2_ transitions, respectively. The Fe 2p spectrum confirms that iron exists predominantly in the Fe^3+^ oxidation state in both CoFe_2_O_4_ and BiFeO_3_ phases. The absence of Fe­(II) suggests that the synthesis conditions promoted the formation of fully oxidized Fe­(III) species, which is consistent with literature reports on CoFe_2_O_4_ spinel ferrites. Finally, the uniformly single XPS Gaussian peak of O 1s (531.3 eV) is assigned to metal–oxygen bonds (M–O) in the spinel CoFe_2_O_4_ and perovskite BiFeO_3_ structures. Thus, we can conclude that the XPS analysis confirms the successful synthesis of the CoFe_2_O_4_@BiFeO_3_ core–shell structure with the expected oxidation states: Fe­(III) in both phases, Co­(II) in CoFe_2_O_4_, and Bi­(III) in BiFeO_3_. The chemical shifts in the core-level spectra are minimal, suggesting a well-formed interface between the two materials without significant interdiffusion.

**Figure 2:**
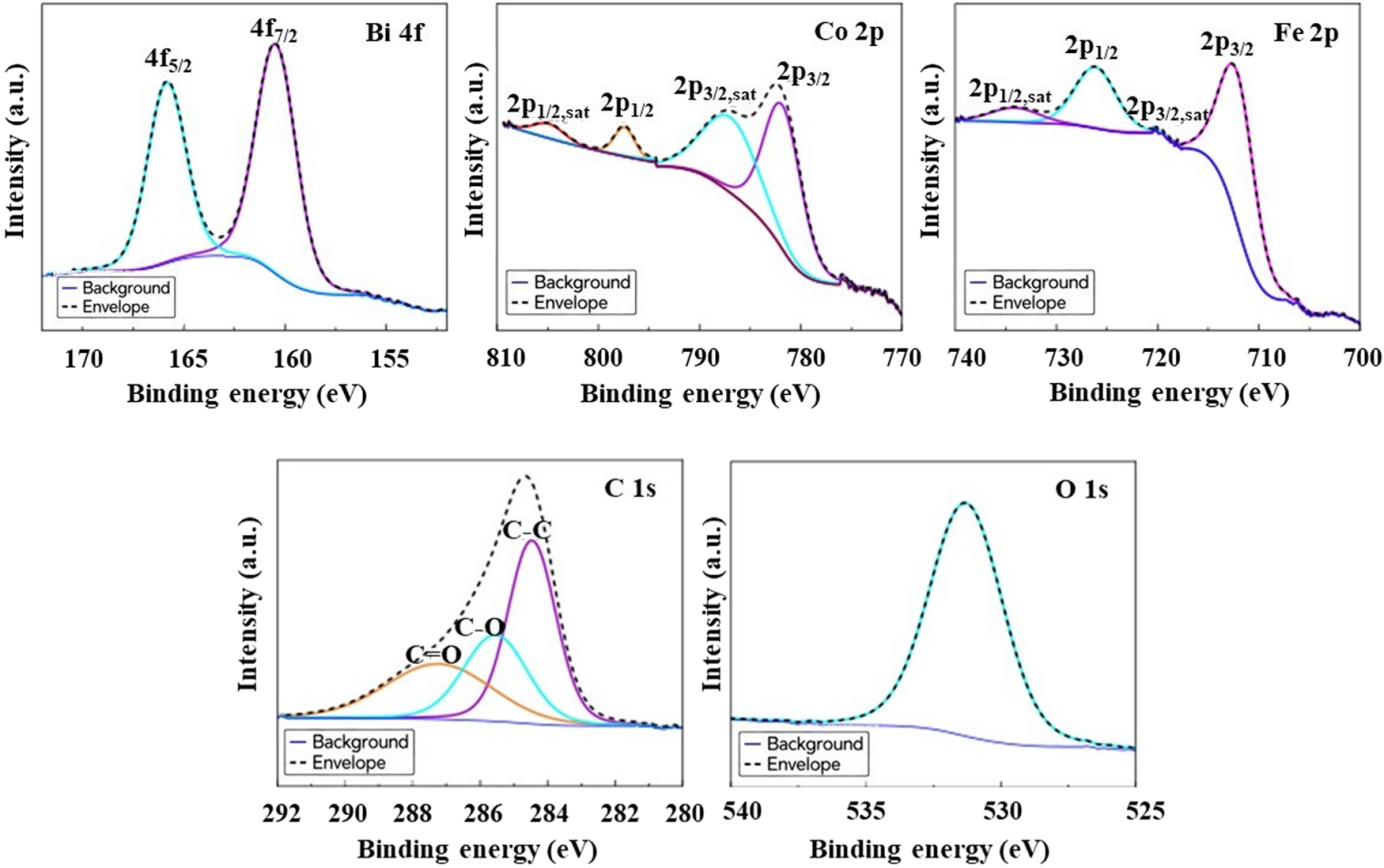
High-resolution XPS spectra of Bi 4f, Co 2p, Fe 2p, C 1s and O 1s recorded acquired from CoFe_2_O_4_@BiFeO_3_ NPs.

### Magnetic Properties

The hysteresis loops corresponding to CoFe_2_O_4_ core and CoFe_2_O_4_@BiFeO_3_ core–shell NPs are shown in Figure 3A. As revealed by TEM, the observed particle size range exceeds the typical superparamagnetic threshold for CoFe_2_O_4_-based nanoparticles, which is consistent with the presence of hysteresis and nonzero coercivity. Therefore, the nanoparticles exhibit ferrimagnetic behavior rather than superparamagnetism under the studied conditions. The values of saturation magnetization (*M*_s_) and coercivity (*H*_c_) for the core are similar to those reported for CoFe_2_O_4_ nanostructures in the literature, with a tendency for *M*_s_ and *H*_c_ to increase after annealing.^37^ Although thermal annealing can promote nanoparticle coarsening and partial agglomeration, it also enhances crystallinity and reduces structural defects, which contributes to the observed increase in saturation magnetization. Furthermore, the higher coercivity observed in CoFe_2_O_4_ @BiFeO_3_ nanoparticles compared to CoFe_2_O_4_ alone is attributed to interfacial exchange coupling between the ferrimagnetic core and the antiferromagnetic shell, which increases magnetic anisotropy and resistance to magnetization reversal. Table 1 summarizes the *M*_s_ and *H*_c_ values for the nonannealed and annealed CoFe_2_O_4_ core and CoFe_2_O_4_@BiFeO_3_ core–shell NPs. Interestingly, the coercivity in CoFe_2_O_4_@ BiFeO_3_ ME NPs is larger than for pure CoFe_2_O_4_, especially after annealing, which is a possible indication of ferrimagnetic/antiferromagnetic exchange interactions between the CoFe_2_O_4_ core and the BiFeO_3_ shell [30]. Annealing increases *H*_c_ of CoFe_2_O_4_ NPs by 13.5%, but the effect of annealing on *H*_c_ in CoFe_2_O_4_@BiFeO_3_ NPs is a 108% increase, indicating the critical role of BiFeO_3_ in inducing such magnetic hardening effect. Larger *H*_c_ values have indeed been reported in exchange-coupled CoFe_2_O_4_@BiFeO_3_ NPs compared to CoFe_2_O_4_ NPs.^38^ This effect is ascribed to the microscopic interfacial torque that BiFeO_3_ spins exert on the CoFe_2_O_4_ core magnetic moment upon magnetization reversal. It is a common manifestation of exchange interacting ferromagnetic (or ferrimagnetic)–antiferromagnetic systems.^39^ Further experimental evidence of this exchange bias coupling can be obtained by cooling the core–shell NPs from a temperature slightly above the BiFeO_3_ Néel temperature (*T*_N_ = 640 K) under a constant magnetic field (*H*_FC_), which should result in a shift of the hysteresis loop along the magnetic field axis. We field-cooled the ME NPs from 650 K while applying *H*_FC_ = 10 kOe. The results are shown in Figure 3B. While the hysteresis loop before field cooling is rather symmetric (with a *H*_c_ value of 2170 Oe), a small offset of the loop toward negative fields is observed after field cooling, with a net exchange bias field of 26 Oe, which is a typical value for this system.^40^ This indicates that CoFe_2_O_4_ and BiFeO_3_ are in intimate contact, a condition which is important for the occurrence of magnetoelectric effects in this system. Moreover, these results would not be expected if such interfaces were present only in isolated or nonreproducible nanoparticles, confirming once again the correct-core–shell formation.

**Figure 3:**
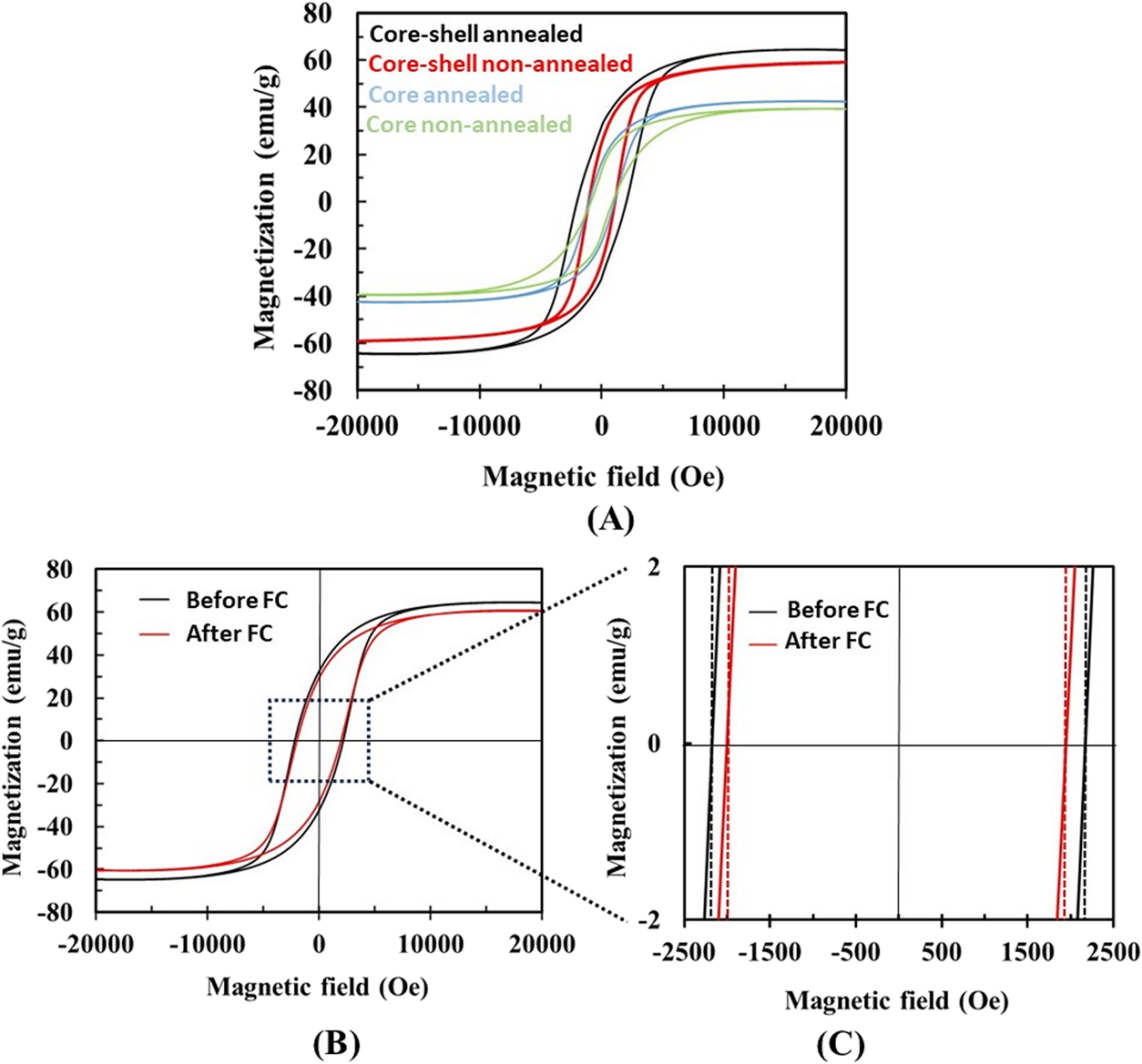
Magnetic characterization of CoFe_2_O_4_ core and CoFe_2_O_4_@ BiFeO_3_ core–shell NPs. (A) Room-temperature vibrating sample magnetometry (VSM) hysteresis loops of CoFe_2_O_4_ core and CoFe_2_O_4_@ BiFeO_3_ core–shell NPs, measured in both nonannealed and annealed states. (B) VSM hysteresis loops of the CoFe_2_O_4_@ BiFeO_3_ core–shell NPs recorded before and after field cooling (FC). (C) Enlarged view of the central region of the hysteresis loops in (B).

**Table 1:** Saturation Magnetization (*M*_*s*_) and Coercivity (*H*_*c*_) Values for the CoFe_2_O_4_ Core and CoFe_2_O_4_@ BiFeO_3_ Core-Shell NPs both in Non-Annealed and Annealed States

property/hydrogel	CoFe_2_O_4_ non annealed	CoFe_2_O_4_ annealed	CoFe_2_O_4_@BiFeO_3_ nonannealed	CoFe_2_O_4_@BiFeO_3_ annealed
*M*_s_ (emu g^–1^)	41 ± 1	59 ± 1	42 ± 1	65 ± 1
*H*_c_ (Oe)	980 ± 4	1112 ± 3	1041 ± 3	2171 ± 4

### Characterization of the GelMA-ME NPs Hydrogels

First, GelMA was synthesized involving the functionalization of gelatin with methacrylic anhydride through covalent modification of its amine-containing residues, primarily lysine, forming a covalent amide linkage (−NHCOCH = CH_2_) (see scheme in Figure S5). In order to confirm the synthesis of GelMA and its degree of methacrylation (DoM) ^1^H NMR spectroscopy was performed. The gelatin and the GelMA ^1^H NMR spectra were compared (Figure S5). Specific peaks associated with the methacrylate groups introduced into the gelatin backbone were identified. New proton signals appeared between δ 5.4 and 5.6 ppm and at δ 1.9 ppm in the spectrum of GelMA, which were attributed to the methacrylate vinyl protons (−C **H**_**2**_=C­(CH_3_)−) and methyl protons (−CH_2_=C­(C **H**_**3**_)−), respectively. Additional small signals between δ 6.1 and 5.7 ppm for methacrylate vinyl protons were also observed. Those new peaks confirm the successful grafting of methacrylate groups onto gelatin. On the other hand, the peak of the free lysine (NH_2_–C **H**_2_–CH_2_–CH_2_–CH_2_−) of the unmodified gelatin at δ 3 ppm markedly decreased in GelMA, indicating once again that the methacrylic group was successfully bound to the gelatin. The reduction in the integrated signal was utilized to determine the DoM, as the primary amine of lysine serves as the primary reaction site. The calculated DoM is around 96%, which is similar to other reported values for GelMa.^41^

After that, GelMA-ME NPs hydrogels were easily prepared by homogeneously mixing GelMA and core–shell ME NPs followed by the subsequent photopolymerization of the methacryloyl groups using UV irradiation leading to the chemical cross-linking of GelMA. Bare GelMa hydrogels were also prepared for comparison purposes. The morphology of the GelMA and hybrid GelMA-ME NPs hydrogels was evaluated using SEM Figure 4A shows an image of the cross-section where a highly porous and open structure can be observed. Moreover, the hydrogel shows interconnectivity among the different porous, which is of interest for tissue engineering applications. On the other hand, round lighter particles homogeneously distributed on the polymeric matrix can be detected, which were attributed to the ME NPs. It is also important to highlight that the incorporation of the NPs into the hydrogel matrix did not alter the microstructure of the bare GelMA hydrogel. Moreover, the NPs presence did not seem to disrupt the continuity of the polymeric matrix, allowing the formation of a continuous and stable composite hydrogel. Energy dispersive X-ray spectroscopy (EDX) analysis of the hydrogel cross-section confirmed the successful incorporation of the ME NPs into the hydrogel as Bi, Co and Fe signals were detected (Figure 4B). The porosity of the hydrogels was evaluated through micro-CT (Figure 4C). A decrease in the mean pore size, from around 47 μm to approximately 29 μm, was observed when the ME NPs were incorporated in the GelMA hydrogel. This indicates that the presence of the ME NPs blocks the pores in the GelMA hydrogel.

**Figure 4:**
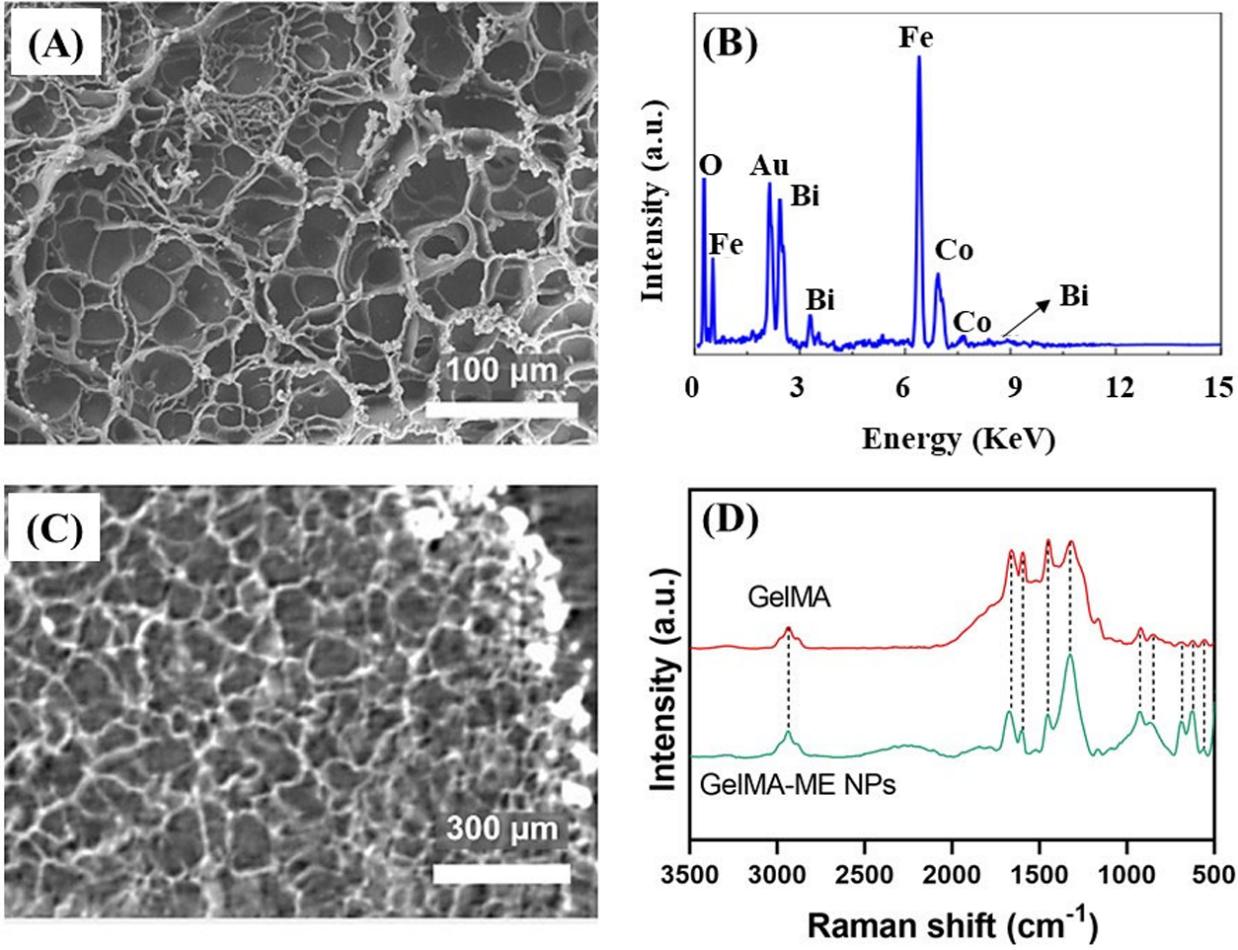
(A) SEM image, (B) EDX spectrum, and (C) Micro-CT image of the GelMA-ME NPs hydrogel. (D) Raman spectra of the GelMA and GelMA-ME NPs hydrogels.

In this line, the equilibrium swelling behavior of the hydrogels was quantitatively evaluated to probe their water uptake capacity and infer changes in network structure through the determination of the swelling ratio (SR). Prior to measurement, the hydrogels were lyophilized and subsequently immersed in phosphate-buffered saline (PBS) for 24 h to ensure equilibrium swelling conditions. The SR was calculated from the mass difference between the dry and swollen states. The pristine GelMA hydrogel exhibited a swelling ratio of 61%, whereas the GelMA-ME NPs nanocomposite hydrogel showed a reduced swelling ratio of 49%. According to the Flory–Rehner theory, this decrease in swelling is indicative of an increased effective cross-linking density and reduced polymer chain mobility. This interpretation is supported by the micro-CT results, which revealed a more compact network architecture and reduced pore size in the presence of ME NPs. Importantly, these structural changes also correlate well with the compression testing results, as the GelMA-ME NPs hydrogels exhibited an increased compressive modulus compared to pristine GelMA. The combined reduction in swelling and enhancement in compressive stiffness suggests that ME NPs act as physical cross-linking centers and/or promote additional chain entanglements, thereby restricting solvent diffusion and limiting hydrogel expansion but reinforcing the hydrogel network and improving load-bearing capability.

Raman spectroscopy was performed on the hydrogels in order to elucidate their chemical structure. Figure 4D shows the spectra of GelMA and GelMA-ME NPs hydrogels. Both spectra show several intense peaks in the midwavenumber region (1000–1800 cm^–1^) corresponding to C = O stretching (Amide I) and N–H bending and C–N stretching (Amide II) of peptide bonds in GelMA as well as the C = C stretching peak from methacrylate groups, which is indicative of GelMA polymerization. Moreover, some other transitions are observed in the high-wavenumber region (2800–3500 cm^–1^) attributed to O–H stretching (3200–3500 cm^–1^), which corresponds to hydrophilic hydroxyl groups, and to C–H stretching (∼2800–3000 cm^–1^) from aliphatic methylene (−CH_2_) and methyl (−CH_3_) groups in GelMA. While the low wavenumber region (200–1000 cm^–1^) is mostly featureless for GelMA hydrogel, as it does not have characteristic vibrational modes in this range, many strong peaks are observed in the hybrid hydrogel. Those peaks are attributed to the metal–oxygen vibrations of the CoFe_2_O_4_ core (Fe–O, Co–O) and BiFeO_3_ shell (Fe–O, Bi–O). On the other hand, some changes in the midwavenumber region are detected like a decrease in peak intensity for Amide I and Amide II and their shift, which may indicate possible coordination interactions between ME NPs and GelMA functional groups. Thus, we can confirm the successful integration of the ME NPs into the GelMA hydrogel and their interaction.

The mechanical properties of the GelMA and GelMA-ME NPs hydrogels were evaluated via compression tests. Figure 5 shows the stress–strain curves for both hydrogels and Table 2 the corresponding tensile strength and Young modulus (*E*) values. Although both curves are qualitatively similar, quantitative differences in the maximum strength and the stiffness are observed. Thus, maximum strength, which was defined as the stress at 75% strain, is slightly higher for the GelMA-ME NPs hydrogel than for the bare GelMA indicating the reinforcing effect of the NPs. It is also important to remark that both hydrogels can be compressed to a height of 75% of the initial cylindrical samples without fracturing. On the other hand, compressive *E* modulus, which would be a key indicator of the hydrogel’s ability to accommodate the compressive stress of the cardiac beat, exhibits a significantly higher value for GelMA-ME NPs hydrogel than for GelMA. This higher stiffness is attributed not only to the interaction between the polymer and ME NPs as we observed by Raman, but also to the presence of the ME NPs themselves with high stiffness and originating entanglement of the polymeric chains and therefore reducing the flexibility of the hydrogel’s matrix. The obtained values are in agreement with those found in the literature for similar GelMA-based hydrogels.^42^ Finally, it is worthy to remark that hydrogel’s Young modulus values are slightly lower than the stiffness of native human myocardium (10–15 kPa).^43^ However, cardiac patches with similar Young’s moduli have been reported to support cardiomyocyte adhesion, contractility, and mechanosensitive signaling of cultured cardiomyocytes more effectively than highly stiff materials.^44–46^ Finally, while the compressive stress at high strain (∼75%) provides useful information about hydrogel mechanical robustness and resistance to deformation, it does not directly correspond to physiological cardiac strains, which are typically smaller during the cardiac cycle. Instead, this parameter should be interpreted as an indicator of structural integrity and durability of the hydrogel network rather than a direct biomimetic comparison with myocardial mechanics.

**Figure 5:**
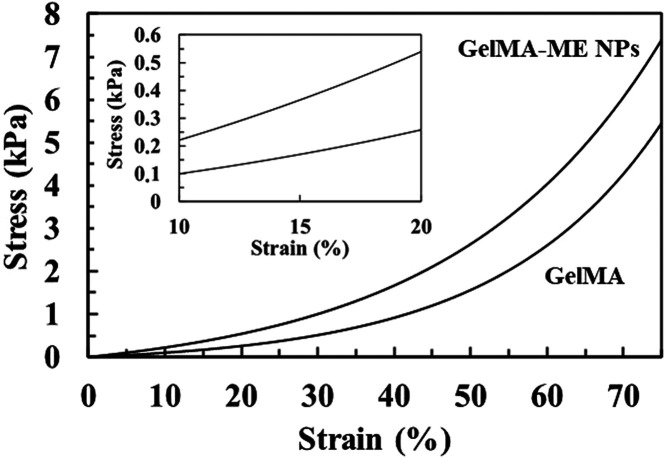
Compressive stress–strain curves for GelMa and GelMA-ME NPs hydrogels. Inset is an enlarged view of the 10–20% strain region used for compressive elastic modulus calculation.

**Table 2:** Maximum Stress and Compressive Elastic Modulus Values Obtained from the Stress-strain Curves in Figure 5 ^a^

	GelMA	GelMA-ME NPs
maximum stress (kPa)	6.4 ± 0.9^(a)^	6.7 ± 0.9^(a)^
elastic modulus (kPa)	1.8 ± 0.6^(a)^	3.5 ± 0.4^(b)^

a Different letters (a,b) indicate statistically significant differences (*p* < 0.05) between conditions.

### Biological Characterization

Cell viability within GelMA and GelMA-ME NPs hydrogels was qualitatively assessed by Live/Dead assay over a 10 day-period of culture under both unstimulated conditions and wireless magnetic stimulation (150 mT, 500 Hz). These stimulation parameters were selected based on previous studies and considering the magnetic response of the CoFe_2_O_4_@BiFeO_3_ NPs. In particular, the applied field amplitude is of the same order of magnitude as the coercive field of the NPs, enabling efficient magnetostrictive deformation of the CoFe_2_O_4_ core and strain transfer to the BiFeO_3_ shell, which is essential for magnetoelectric coupling.^15^ The selected frequency lies within the range commonly used for dynamic magnetoelectric stimulation, allowing repeated actuation while avoiding excessive heating or mechanical damage to cells.^16,47–49^ Additionally, the use of a rotating permanent-magnet system minimizes heating, ensuring that the observed biological responses arise predominantly from magnetoelectric effects.

Representative fluorescence microscopy images are shown in Figure 6. Under no stimulation conditions, both GelMA and GelMA-ME NPs hydrogels exhibited a high proportion of viable cells at all-time points, with minimal red fluorescence, confirming the intrinsic cytocompatibility of both hydrogel formulations. At day 1, cells were homogeneously distributed within both matrices, indicating efficient adhesion and encapsulation. Over time, an increase in cell density was observed, particularly by day 10, consistent with the progressive increase in metabolic activity measured by the PrestoBlue assay (see below). Notably, GelMA-ME NPs hydrogels displayed a higher cell density and more organized cellular morphology at later time points, with cells exhibiting increased spreading, elongation, and interconnection suggesting strong adhesion to the hydrogel surface and a favorable microenvironment for cell growth (Figure 6, and Figure S6). This behavior may reflect nanoparticle-mediated modulation of matrix stiffness and cell-matrix interactions. These results confirm the excellent biocompatibility of both hydrogel types toward the H9c2 cells over the cultured period with high potential for cell culture and heart tissue engineering.

**Figure 6:**
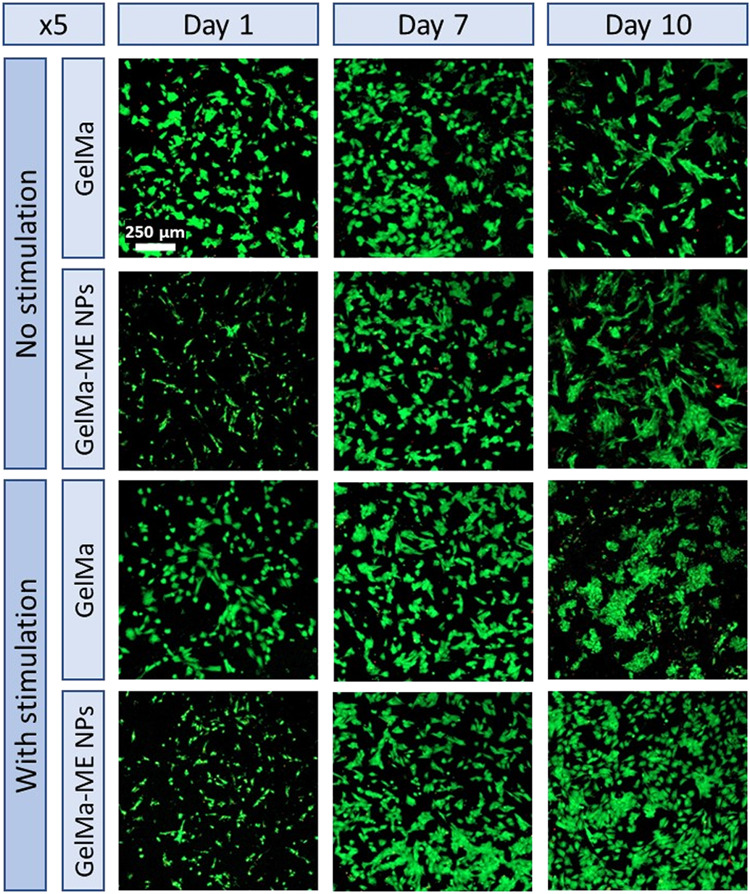
Live/Dead fluorescence staining of H9c2 cells cultured on GelMA and GelMA-ME NPs hydrogels under no stimulation and with magnetic stimulation at days 1, 7, and 10 (×5 magnification). Live cells are shown in green and dead cells in red.

Upon wireless electric stimulation through the application of a dynamic magnetic field, cell density increased across all samples, confirming that the applied stimulation parameters do not induce cytotoxic effects. Notably, a substantial increase in live cell density and surface coverage was observed in the GelMA-ME NPs hydrogels compared to stimulated GelMA alone. By day 7, GelMA-ME NPs hydrogels exhibited a visibly higher cell density under magnetic stimulation, which further increased by day 10. The morphological features observed in the stimulated GelMA-ME NPs hydrogels are particularly relevant. Cells exhibited enhanced spreading, elongation, and formation of interconnected networks, which are hallmarks of cardiomyocyte maturation and functional coupling. Such morphological organization is critical for the development of electrically synchronized cardiac tissue, as cell alignment and connectivity directly influence action potential propagation and contractile behavior. The weaker response observed in pristine GelMA hydrogels under stimulation suggests that magnetic fields alone are insufficient to elicit robust bioelectrical responses in the absence of an active transduction mechanism due to the nonelectrically conductive nature of pristine GelMA. Therefore, the observed stimulation does not arise from bulk electronic conduction through the hydrogel. Instead, the embedded ME CoFe_2_O_4_@BiFeO_3_ NPs within the hydrogel act as local transducers, generating localized electric fields at the nanoparticle-cell interface under an external magnetic field. The hydrogel matrix serves as a hydrated and ionically conductive environment that facilitates the interaction between cells and these localized electric cues. These bioelectrical cues are known to promote cell spreading, cytoskeletal organization, and proliferation through modulation of membrane potential, calcium ion influx, and downstream signaling pathways.^8–10^ Finally, it is also worthy remarking that the spatial homogeneity of live cells throughout the hydrogels suggests that the wireless stimulation effect is not limited to the hydrogel surface, but rather propagates throughout the 3D construct, highlighting a key advantage of magnetoelectric nanocomposites over conventional electrode-based stimulation approaches.

In order to quantitatively evaluate proliferation, cell metabolic activity within GelMA and GelMA-ME NPs hydrogels was evaluated over a 10-day culture period using the PrestoBlue assay under both unstimulated conditions and magnetic stimulation (150 mT, 500 Hz). Under no stimulation conditions, both hydrogel formulations supported a progressive increase in metabolic activity over time, indicating good cytocompatibility and sustained cell proliferation (Figure 7). At early time points (days 1 and 3), no substantial differences were observed between GelMA and GelMA-ME NPs, suggesting that the incorporation of ME NPs does not adversely affect initial cell viability or early metabolic activity. From day 7 onward, GelMA-ME NPs exhibited a moderately higher metabolic activity compared to pristine GelMA, reaching a statistically significant difference by day 10 (****, *p* < 0.0001). This enhanced metabolic response may be attributed to subtle changes in the microenvironment induced by the ME NPs, such as increased matrix stiffness, altered pore architecture, or improved cell–matrix interactions, as suggested by the swelling and mechanical characterization.

**Figure 7:**
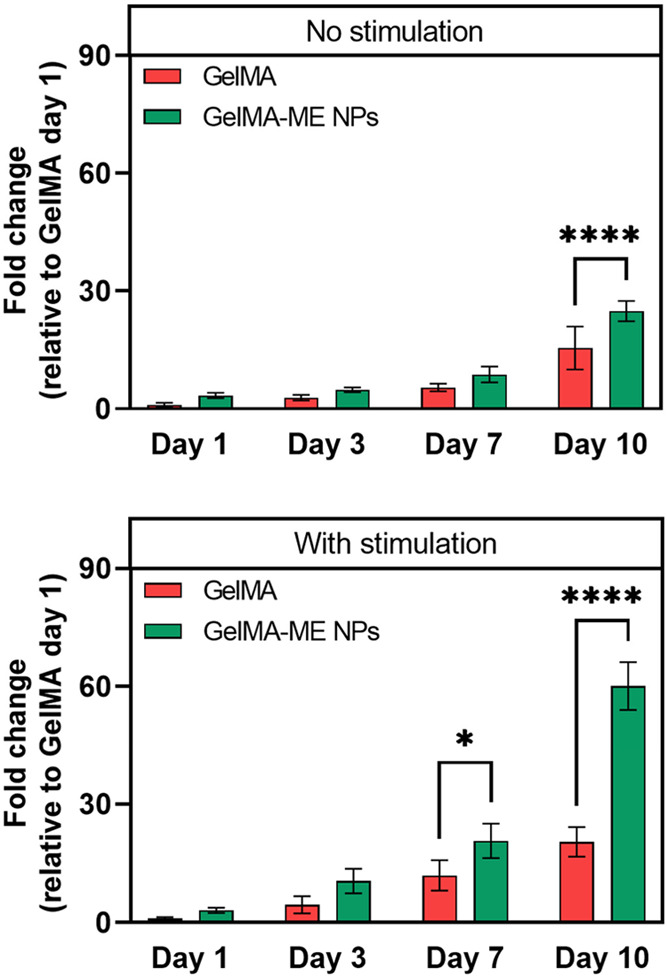
Quantification of H9C2 cell proliferation on GelMA and GelMA-ME NPs hydrogels under no stimulation and with magnetic stimulation at days 1, 3, 7, and 10, measured by the PrestoBlue assay. Data are expressed as fold change relative to GelMA at day 1. Asterisks indicate statistically significant differences (*p < 0.05, ****p < 0.0001).

Under magnetic stimulation, a more pronounced enhancement in metabolic activity was observed for both systems, with a markedly stronger effect in the GelMA-ME NPs hydrogels (Figure 7). While GelMA hydrogels showed a gradual increase in metabolic activity over time, the GelMA-ME NPs hydrogels exhibited a significantly accelerated and amplified response, particularly at later culture times. By day 10, the metabolic activity of cells encapsulated in GelMA-ME NPs under stimulation was approximately 3-fold higher than that of GelMA hydrogels (****, *p* < 0.0001) and significantly higher than all unstimulated conditions. The synergistic effect observed in the GelMA-ME NPs hydrogels under magnetic stimulation can be attributed to the magnetoelectric nature of the embedded nanomaterials. Upon exposure to an alternating magnetic field, ME NPs are expected to generate localized electric cues within the hydrogel matrix, thereby providing wireless electrical stimulation to the encapsulated cells. Such bioelectrical signals are known to enhance cellular metabolic activity, proliferation, and signaling pathways associated with electrostimulation. In contrast, pristine GelMA lacks the ability to transduce magnetic stimuli into electrical signals, explaining the comparatively weaker response under stimulation. Importantly, the absence of cytotoxic effects at early time points and the sustained increase in metabolic activity over 10 days confirm the cytocompatibility of both the nanocomposite hydrogel and the applied magnetic stimulation regime. Thus, these results demonstrate that the integration of ME NPs into GelMA hydrogels function as effective platforms for remote, noninvasive modulation of cellular activity, significantly enhancing cell metabolic performance under magnetic stimulation while maintaining favorable baseline biocompatibility, offering significant potential for applications in tissue engineering and regenerative medicine.

Overall, the Live/Dead and the PrestoBlue results demonstrate that GelMA-ME NPs hydrogels provide a cytocompatible and electrically active microenvironment capable of supporting enhanced cell viability, proliferation, and cardiac-relevant morphological organization under wireless electric stimulation through the application of a magnetic field. These features underscore the potential of magnetoelectric nanocomposite hydrogels as advanced platforms for engineered cardiac tissues and implantable or wearable wireless cardiac bioelectronic systems. It should be noted that direct measurements of the magnetoelectric coefficient were not performed in this study. The magnetoelectric behavior is inferred from the structural characterization, magnetic coupling (including exchange bias), and the enhanced biological response under magnetic stimulation. Future work will focus on direct quantification of magnetoelectric output and systematic optimization of magnetic field parameters.

## Conclusions

In this study, we have successfully developed magnetoelectric GelMA-based hydrogels that combine the intrinsic bioactivity, biocompatibility and softness of gelatin-derived matrices with the unique ability to deliver wireless electrical stimulation through ME NPs transduction. The integration of CoFe_2_O_4_@BiFeO_3_ core–shell NPs within a photo-cross-linked GelMA network yields porous, mechanically compliant, and cytocompatible 3D scaffolds capable of converting external magnetic fields into localized electric cues at the cellular scale. The nanocomposite hydrogels exhibit reduced swelling and enhanced mechanical robustness while maintaining a Young’s modulus compatible with cardiac tissue. Importantly, under dynamic magnetic stimulation, the magnetoelectric hydrogels significantly promote cardiac cell proliferation, metabolic activity, and structural organization, outperforming nonmagnetoelectric GelMA controls and demonstrating the necessity of active magnetoelectric coupling. Although direct quantification of the magnetoelectric coefficient was not performed in this study, the presence of interfacial exchange coupling, together with prior reports on CoFe_2_O_4_@BiFeO_3_ systems, indeed supports the occurrence of magnetoelectric transduction. The enhanced cellular response under magnetic stimulation further provides indirect functional evidence of electrically mediated stimulation. Our findings confirm that magnetic fields alone are insufficient to drive bioelectrical responses and highlight the central role of ME NPs as local electromechanical transducers. The developed nanocomposite hydrogels overcome key limitations of conventional electrode-based systems by enabling remote, noninvasive, and spatially distributed electrostimulation within soft 3D matrices. This platform offers a versatile and scalable strategy for engineered cardiac tissues, with potential extension to other electrically responsive tissues such as skeletal muscle and neural constructs. Overall, this work establishes magnetoelectric hydrogels as a promising foundation for wireless cardiac regeneration and future bioelectronic therapies.

## Supplementary Material



## Notes

Fluorescence calibration curve to get the number of cells, particle size distribution histogram, TEM image of core–shell ME NPs, HRTEM and FFT analysis of core–shell ME NPs, reaction scheme for GelMA synthesis, ^1^H NMR spectra of gelatin and GelMA, and live/dead fluorescence staining confocal images (PDF)

## Notes

BioRobotics Institute and the Department of Excellence in Robotics, & AI Santa’Anna School of Advanced Studies, Piazza Martiri della Libertà, 33, Pisa, PI, 56127 Italy
