# Supplementary material for: Three-Dimensional Magnetoelectric Nanocomposite GelMA Hydrogels for Wireless Electrical Stimulation of Cardiac Cells
Source: ACS Appl Mater Interfaces. 2026 May 7;18(19):28020–32. doi: 10.1021/acsami.6c05467 (PMC13195578; doi:10.1021/acsami.6c05467)
Supplement: Supplementary file 1 [file am6c05467_si_001.pdf]

Supporting Information

Three-Dimensional Magnetoelectric Nanocomposite GelMA Hydrogels for Wireless Electrical Stimulation of Cardiac Cells

Angel Viteri,[a,b,#,*] Carolina Vargas-Estevez,[a,b,#,†] Samuele Colombi,[b,c] Leonor Resina,[b,c] Huan Tan,[d,e] Jordi Sort,[d,e,f] Maria-Pau Ginebra,[a,b,h,i] Elisabeth Engel,[g,h,i] Carlos Alemán,[b,c,h] Jose García-Torres[a,b,i,*]

[a] *Biomaterials, Biomechanics and Tissue Engineering Group, Department of Materials Science and Engineering and Institute for Research and Innovation in Health (IRIS), Universitat Politècnica de Catalunya-BarcelonaTech (UPC), Av. Eduard Maristany 16, 08019, Barcelona, Spain*

[b] *Barcelona Research Center in Multiscale Science and Engineering, Universitat Politècnica de Catalunya-BarcelonaTech (UPC), Av. Eduard Maristany 16, 08019, Barcelona, Spain*

[c] *Departament d'Enginyeria Química, EEBE, Universitat Politècnica de Catalunya, C/ Eduard Maristany, 10-14, 08019, Barcelona, Spain*

[d] *Departament de Física, Universitat Autònoma de Barcelona, Bellaterra 08193, Spain*

[e] *Catalan Institute of Nanoscience and Nanotechnology (ICN2), CSIC and BIST, Bellaterra 08193, Spain*

[f] *Institució Catalana de Recerca i Estudis Avançats (ICREA), Pg. Lluís Companys 23, Barcelona 08010, Spain.*

[g] *IMEM-BRT group, Department of Materials Science and Engineering, Universitat Politècnica de Catalunya-BarcelonaTech (UPC), 08019, Barcelona, Spain*

[h] *Institute for Bioengineering of Catalonia (IBEC), Barcelona Institute of Science and Technology (BIST), Baldiri Reixac 10-12, 08028, Barcelona, Spain*

[i] *Centro de Investigación Biomédica en Red de Bioingeniería, Biomateriales y Nanomedicina (CIBER-BBN), Instituto de Salud Carlos III, 28029, Madrid, Spain.*

Corresponding authors: angel.viteri@upc.edu, jose.manuel.garcia-torres@upc.edu

[#]These authors have equally contributed to this work.

†Current affiliation: BioRobotics Institute and the Department of Excellence in Robotics, & AI Santa'Anna School of Advanced Studies, Piazza Martiri della Libertà, 33, Pisa, PI, 56127 Italy.

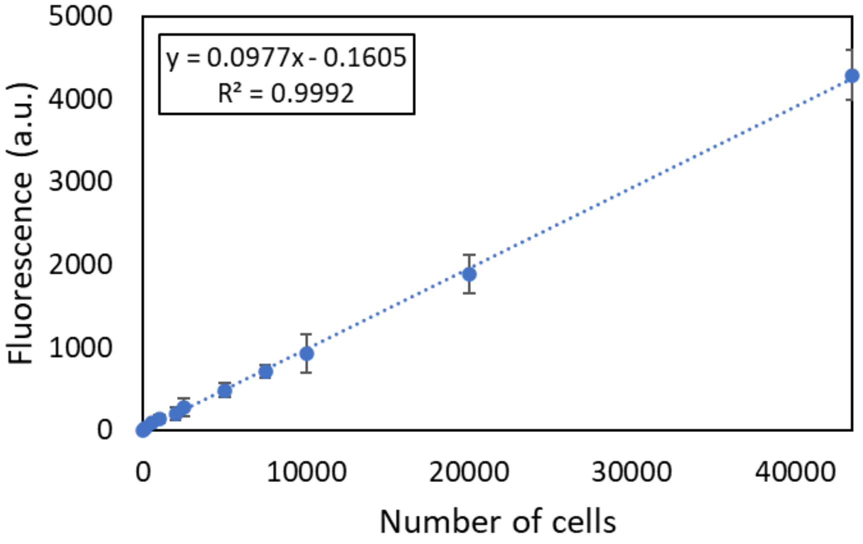

Figure S1. Fluorescence calibration curve obtained by plotting fluorescence intensity against number of cells.

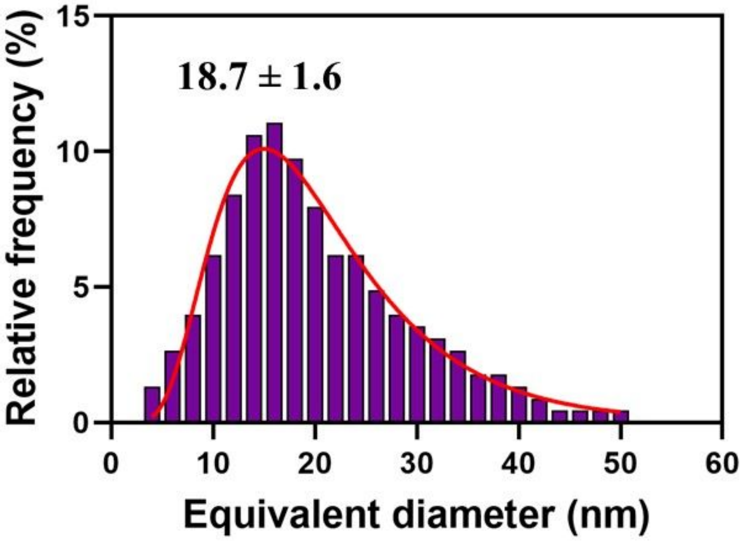

Figure S2. Particle size distribution of ME NPs obtained from TEM image analysis. The histogram shows the relative frequency (%) as a function of the equivalent circular diameter, while the red curve represents the corresponding log-normal fit.

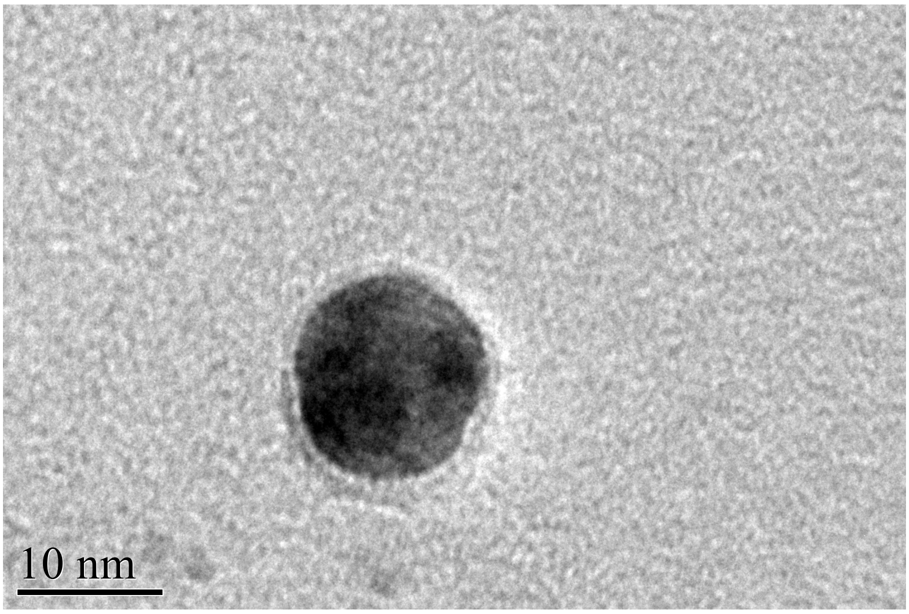

Figure S3. Underfocused TEM image showing the core-shell formation.

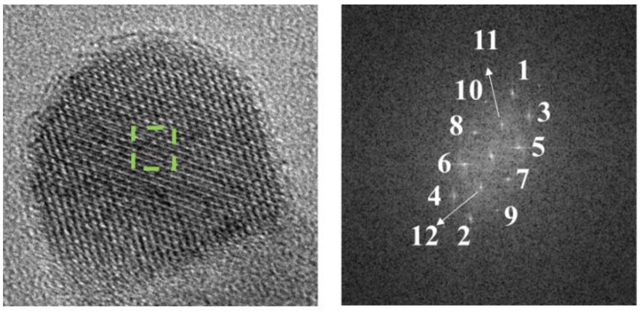

Peak	d-spacing	Plane	Material
1,2	0.2118	(400)	$CoFe_2O_4$
3,4	0.2553	(311)	$CoFe_2O_4$
5,6	0.4867	(111)	$CoFe_2O_4$
7,8	0.4875	(111)	$CoFe_2O_4$
9,10	0.2542	(311)	$CoFe_2O_4$
11,12	0.3895	(012)	$BiFeO_3$

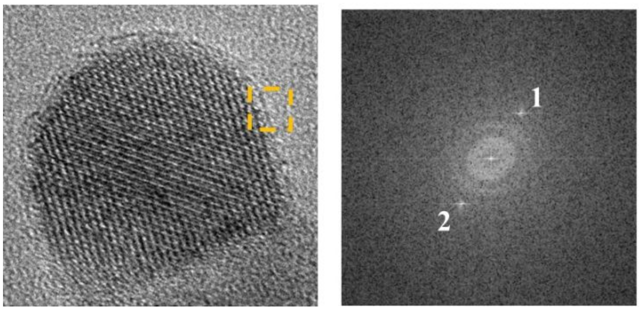

Peak	d-spacing	Plane	Material
1,2	0.3895	(012)	$BiFeO_3$

Figure S4. HRTEM images and corresponding FFT analysis performed in the shell (upper row) and the core (lower row).

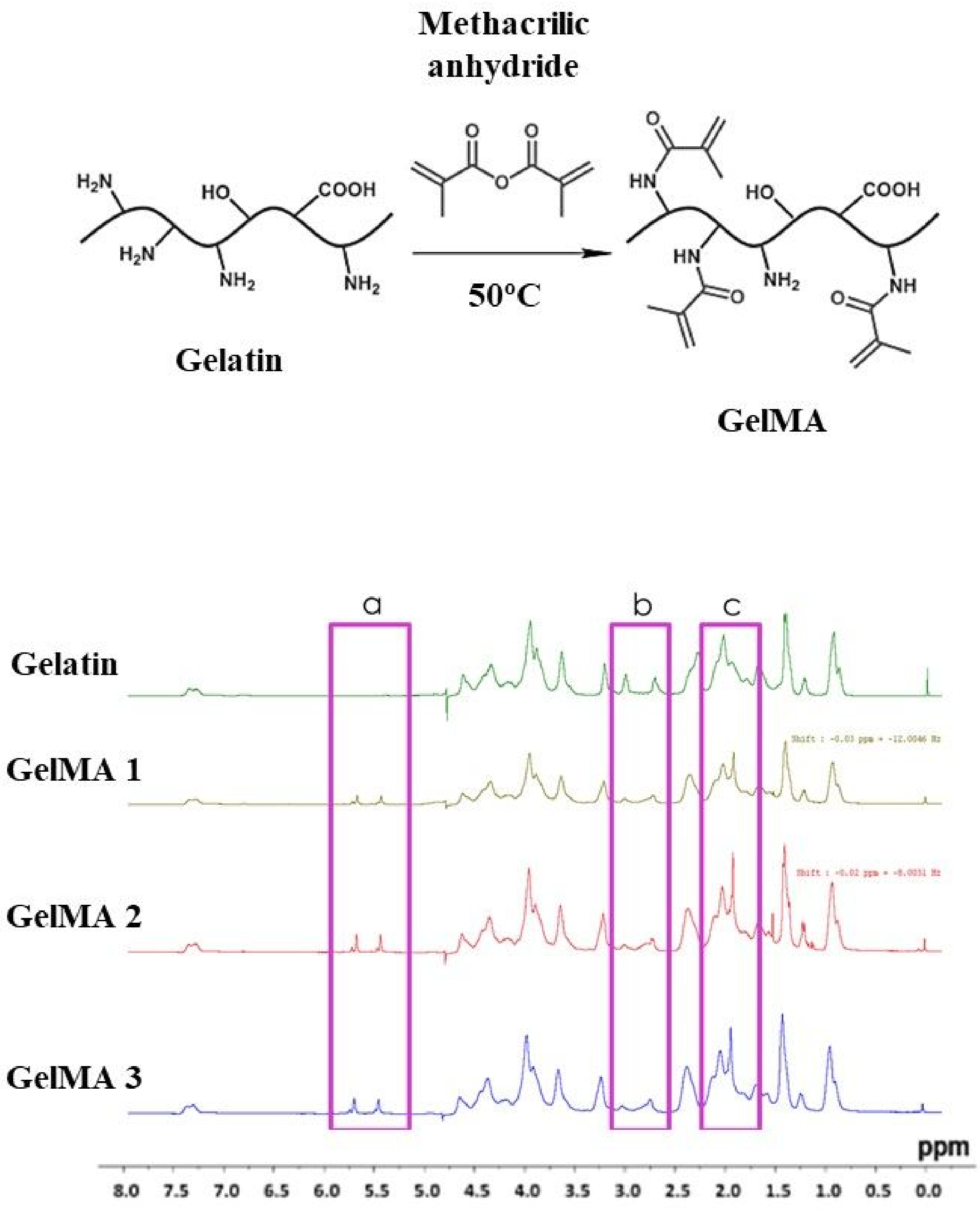

Figure S5. Synthesis of GelMA involving the reaction of gelatin with methacrylic anhydride. ^{1}H-NMR spectra of gelatine and three GelMA samples. Specific regions of gelatin and GelMA were highlighted: a) acrylic protons of metacrylamide groups, b) Signal associated to lysine methylene (used to quantify the DoM), and c) methyl function of methacrylate grafted to gelatin structure.

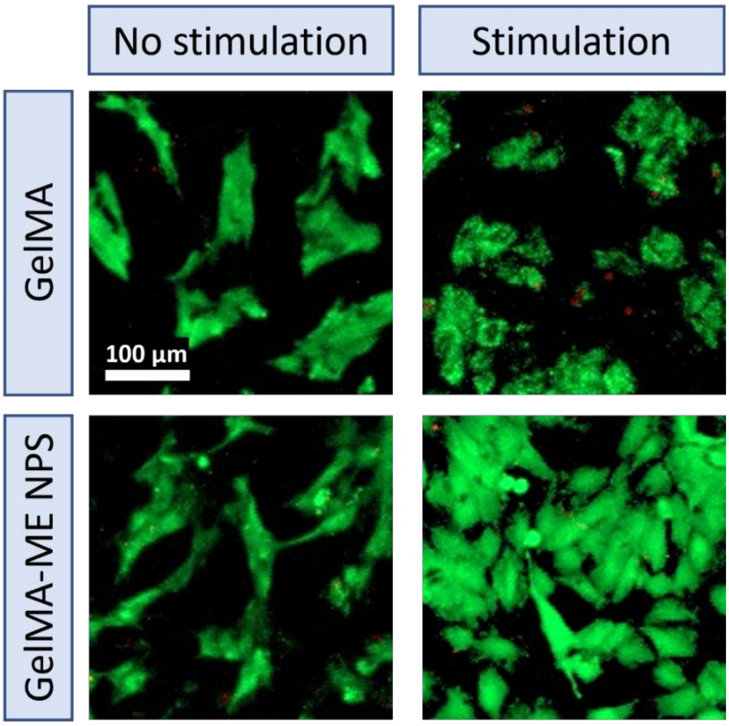

Figure S6. Live/Dead fluorescence staining of H9c2 cells cultured on GelMA and GelMA-ME NPs hydrogels under no stimulation and with magnetic stimulation at day 10 (×10 magnification). Live cells are shown in green and dead cells in red.
